# Causal Association Between Lipid-Lowering Drugs and Glaucoma: A Drug-Targeted Mendelian Randomization Study

**DOI:** 10.1167/iovs.65.10.23

**Published:** 2024-08-14

**Authors:** Yaqiong Liu, Tao Li

**Affiliations:** 1 Regenerative Medicine Institute (REMEDI), University of Galway, Galway, Ireland. E-mail: t.li9@universityofgalway.ie.

We read with interest the meta-analysis by Wang et al.^1^ on the relationship between hyperlipidemia and glaucoma risk. This study provides evidence for the hypothesized role of hyperlipidemia as a risk factor for increased risk of glaucoma (i.e., that hyperlipidemia is associated with elevated intraocular pressure). Interestingly, a case–control study conducted by McGwin et al.^2^ suggests that the use of statin or non-statin cholesterol-lowering medications may be associated with a reduced risk of glaucoma. In addition, two prospective cohort studies reported that long-term statin use appears to be associated with a reduced risk of glaucoma.^3^^,^^4^

However, challenges remain due to isolating remaining confounding variables and addressing limitations inherent in cohort studies, such as demographic, racial, and size differences. Moreover, there is a lack of randomized controlled trials to test the effectiveness of lipid-lowering drugs in glaucoma. In this context, Mendelian randomization (MR) analysis emerges as a useful tool, as it can provide evidence that falls between randomized controlled trials and observational studies.^5^ In this study publicly available data from genome-wide association studies (GWAS) were utilized to explore the genetic association between lipid-lowering drugs and glaucoma.

Three low-density lipoprotein cholesterol (LDL-C)–lowering drugs were included as exposures in this study: 3-hydroxy-3-methylglutaryl-CoA reductase (HMGCR), Niemann–Pick C1-like 1 (NPC1L1), and proprotein convertase subtilisin/kexin type 9 (PCSK9) inhibitors. Single nucleotide polymorphisms (SNPs) located within ±100 kb of HMGCR and PCSK9 loci were used as instrumental variables for these three LDL-C–lowering drugs. The significance level was set at *P* < 5 × 10^− 8^ to extract instrumental variables that had a strong correlation with exposures. To maximize the strength of the instrument, SNPs were allowed to be in low weak linkage disequilibrium (*r*^2^ < 0.30) with each other. GWAS data for LDL were collected from Global Lipids Genetics Consortium (ieu-a-300), which contains 173,082 donors. The GWAS summary statistics for glaucoma (finn-b-H7_GLAUCOMA) were used as outcome and contained 8591 cases and 210,201 controls. All GWAS summary statistics can be sourced from the IEU OpenGWAS project (https://gwas.mrcieu.ac.uk/). The MR analysis was performed using the weighted median and inverse-variance weighted (IVW) methods (Supplementary Tables S1–S12). Cochran's *Q* statistic and the MR-Egger intercept test were utilized to evaluate heterogeneity and horizontal pleiotropy. MR pleiotropy residual sum and outlier (MR-PRESSO) was applied to exclude outliers.

**Figure.**
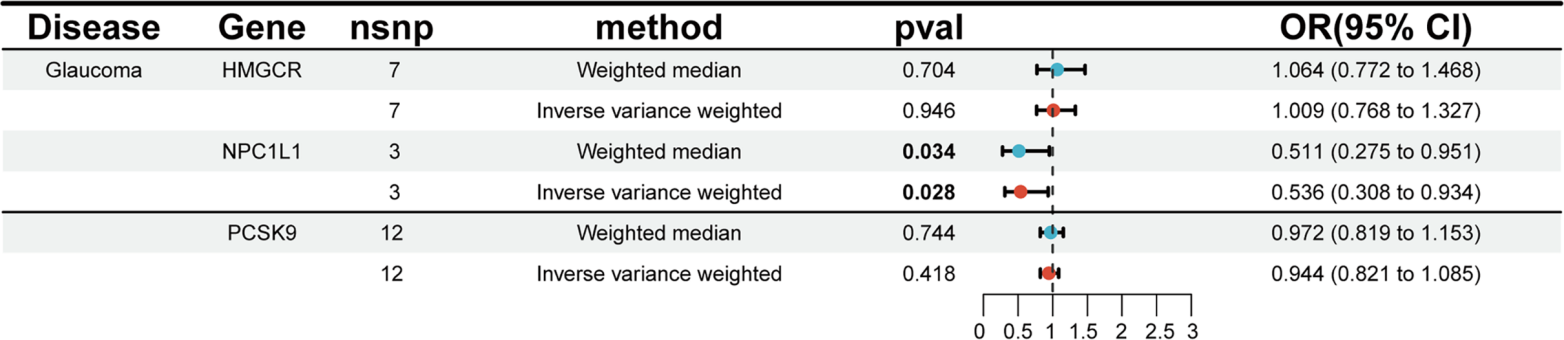
Mendelian randomization analysis of glaucoma with weighted median and IVW method.

The weighted median (odds ratio [OR] = 0.511; 95% confidence interval [CI], 0.275–0.951; *P* = 0.034) and IVW-MR analysis (OR = 0.536; 95% CI, 0.308–0.934; *P* = 0.028) showed that NPC1L1 inhibitors had a significant potential to reduce the risk of glaucoma (Fig. 1). However, HMGCR and PCSK9 inhibitors may not significantly affect the risk of glaucoma (Fig. 1). Additionally, our MR analysis did not show significant heterogeneity or horizontal pleiotropy.

In summary, our drug-targeted MR analysis study suggested that NPC1L1 inhibitors were associated with a decreased risk of glaucoma. This provides additional support for the selection of medications for hyperlipidemic patients with glaucoma.

## Supplementary Material

Supplement 1
